# Auditory Processing in Musicians, a Cross-Sectional Study, as a Basis for Auditory Training Optimization

**DOI:** 10.3390/healthcare11142027

**Published:** 2023-07-14

**Authors:** Maria Kyrtsoudi, Christos Sidiras, Georgios Papadelis, Vasiliki Maria Iliadou

**Affiliations:** 1 Clinical Psychoacoustics Laboratory, 3rd Psychiatric Department, Neurosciences Sector, Medical School, Aristotle University of Thessaloniki, 54124 Thessaloniki, Greeceviliad@auth.gr (V.M.I.); 2 School of Music Studies, Faculty of Fine Arts, Aristotle University of Thessaloniki, 57001 Thermi, Greece

**Keywords:** hearing, auditory processing, cognition, music, byzantine, percussion, rhythm

## Abstract

Μusicians are reported to have enhanced auditory processing. This study aimed to assess auditory perception in Greek musicians with respect to their musical specialization and to compare their auditory processing with that of non-musicians. Auditory processing elements evaluated were speech recognition in babble, rhythmic advantage in speech recognition, short-term working memory, temporal resolution, and frequency discrimination threshold detection. All groups were of 12 participants. Three distinct experimental groups tested included western classical musicians, Byzantine chanters, and percussionists. The control group consisted of 12 non-musicians. The results revealed: (i) a rhythmic advantage for word recognition in noise for classical musicians (*M* = 12.42) compared to Byzantine musicians (*M* = 9.83), as well as for musicians compared to non-musicians (*U* = 120.50, *p* = 0.019), (ii) better frequency discrimination threshold of Byzantine musicians (*M* = 3.17, *p* = 0.002) compared to the other two musicians’ group for the 2000 Hz region, (iii) statistically significant better working memory for musicians (*U* = 123.00, *p* = 0.025) compared to non-musicians. Musical training enhances elements of auditory processing and may be used as an additional rehabilitation approach during auditory training, focusing on specific types of music for specific auditory processing deficits.

## 1. Introduction

Current research provides evidence of enhanced auditory processing in musicians, compared to non-musicians. Capitalizing on this neuroplasticity-based improvement may lead to more focused auditory training for individuals with Auditory Processing Disorder (APD) with the aim of better results and faster rehabilitation. Neuroplasticity, in this case, is the nervous system adaptation resulting from an active response to auditory stimuli. It involves connectivity changes for better performance, especially in related tasks [1]. Hearing is a prerequisite for communication, work, and learning for the average person as well as an essential sense for every musician. Hearing being evaluated by the gold standard pure-tone audiometry may be missing aspects of hearing that are important for everyday life [2]. An audiological evaluation may include speech audiometry as well as tympanometry, stapedial reflexes, otoacoustic emissions, and auditory brainstem responses depending on symptoms and the medical history of a given patient. Communication through the auditory modality needs intact temporal processing, speech in noise perception, working memory, and frequency discrimination [3,4]. Auditory processing happens at the level of the central auditory nervous system. Hearing (i.e., hearing sensitivity and auditory processing) contributes to the formation of cognition, and cognition contributes to hearing [4,5]. The superior auditory processing performance in musicians vs. non-musicians is explained by the enhanced usage and training of their hearing sense, emotion, and listening skills [6]. Musical training goes beyond auditory training to reading and comprehending complex symbols into motor activity [7]. Of interest, recent research shows that frequency precision is more correlated with musical sophistication than cognition [8].

The perception of music and speech is thought to be distinct, although sharing many acoustic and cognitive characteristics [9]. Pitch, timing, and timbre cues may be considered commonalities for auditory information transfer [10]. Memory and attention are required cognitive skills for both music and speech processing. Pitch is the psychoacoustic analogous of the frequency of the sound. Timing refers to specific turning points in the sound (for example, the beginning and the negation of the sound), and timbre is multidimensional and includes spectral and temporal features. Musicians’ superior auditory processing is attributed to enhanced accuracy of neural sound encoding [9,11,12,13] as well as better cognitive function [14,15]. The musical practice embraces the experience of specific sound ingredients as well as joint integration during the performance. Extracting meaning from a complex auditory scene may be a transferable skill to tracking a talker’s voice in a noisy environment [16].

Musicians are in an advantageous position in processing the pitch, timing, and timbre of music compared to non-musicians [17]. They demonstrate strengthened neural encoding of the timbre of their own instrument [18,19,20], but also show enhancements in processing speech [9,21,22,23,24,25] and non-verbal communication sounds [26]. Musical experience promotes a more accurate perception of meaningful sounds in communication contexts other than musical ones [9,12,23,27]. Music training is reported to change brain areas in a specific way that may be predicted by the performance requirements of the specific training instrument [28]. Musicians’ perceptual skills are influenced by the style of music played by them [29,30].

Auditory processing [31] consists of mechanisms that analyze, preserve, organize, modify, refine, and interpret information from the auditory signal. Skills that support these mechanisms are auditory discrimination, temporal and binaural processing, which are known as auditory processing elements. Temporal processing refers to auditory pattern recognition and temporal aspects of audition, divided into four subcomponents: temporal integration, temporal resolution/discrimination (e.g., gap detection), temporal ordering and temporal masking [32]. Sound localization and lateralization and auditory performance with challenging or degraded acoustic signals (including dichotic listening) [33] are included in binaural processing. Auditory discrimination involves the perception of acoustic stimuli in very rapid succession requiring the accuracy of information that is carried to the brain [34,35]. These processes may affect phoneme discrimination, speech in noise comprehension, duration discrimination, rhythm perception, and prosodic distinction [36,37]. Temporal resolution, defined as the shortest period over which the ear can discriminate two signals [38] may be linked to language acquisition and cognition in both children [39,40,41,42] and adults [43,44,45,46].

American Speech Language Hearing Association (ASHA) uses the term Central Auditory Processing Disorder (CAPD) to refer to deficits in neural processing, including bottom–up and top–down neural connectivity [47] of auditory information in the Central Auditory Nervous System (CANS) not as a consequence of cognition or higher order language [33]. Deficits in auditory information processing in the central nervous system (CNS) are demonstrated by poor performance in one or more elements of auditory processing [48]. (C)APD may coexist with, but is not derived from, dysfunction in other modalities. Despite the absence of any substantial audiometric findings, poor hearing and auditory comprehension are expressed in some cases in CAPD. Moreover, (C)APD can be associated with, co-exist or lead to difficulties in speech, language, attention, social, learning (e.g., spelling, reading), learning, and developmental functions [33,49]. In the international statistical classification of diseases and related health problems, 11th edition (ICD-11), auditory processing disorder (APD) is classified as AB5Y as a hearing impairment. (C)APD affects both children and adults, including the elderly [50], and it is linked to functional disorders beyond the cochlea [51,52]. According to WHO [49] prevalence estimates of APD in children range from 2–10% and can affect psychosocial development, academic achievement, social participation, and career opportunities.

### 1.1. Speech Perception in Noise

Speech perception in noise is at the core of auditory processing as the most easily explainable test with a real-life depiction. Temporal elements required to perceive speech may be similar to those needed for music with rhythm thought to stand as a bridge between speech and music [52]. Highly trained musicians have been reported in some studies to have superior performance on different measures of speech in noise [22,52,53,54] with this advantage not always being present [55,56,57].

The consolidating of the possible improved speech in noise perception of musicians may have rehabilitation implications for individuals with hearing impairment [55]. Research outcomes reveal that rhythm perception benefits are present at different levels of speech from words to sentences [58,59]. Percussionists were found to perform relatively better at the sentence in noise level compared to the words in noise one contrasted with vocalists while significantly outperforming non-musicians [52]. There is limited research evaluating speech perception in noise among musicians from different musical styles.

### 1.2. Temporal Resolution

Auditory temporal processing is the alteration of elements of duration within a specific time interval [50]. The ability of the auditory system to respond to rapid changes over time is a component of temporal processing called temporal resolution, linked to stopping consonants perception during a running speech [37,60].

Temporal processes are necessary for auditory processing and the perception of rhythm, pitch, duration, and separating foreground to background [3,36]. Chermak and Musiek [37] highlighted the role of temporal processing across a range of language processing skills, from phonemic to prosodic distinctions and ambiguity resolution. Temporal resolution underlies the discrimination of voiced from unvoiced stop consonants [61] and is clinically evaluated using Gaps-In-Noise [GIN] or Random Gap Detection Test [RGDT] [62]. Evaluating an individual’s ability to perceive a msec gap in noise or between two pure tones provides information on possible deficits in temporal resolution and can lead to a better shaping of rehabilitation [50]. Older adults generally are found to have poorer (longer) gap thresholds than younger adults [5].

Early exposure to frequent music training for years improves timing ability across sensory modalities [63]. Musicians present with better temporal resolution [64,65,66,67,68]. Musicians of different instruments and styles were found to have superior timing abilities compared to non-musicians [65,69]. Longer daily training in music leads to a better gap detection threshold [70]. Neuroplasticity as a result of music training results in enhanced temporal resolution in children that are comparable to adults [66]. To our knowledge, no research publications exist evaluating possible differences in temporal resolution across musicians from different musical styles.

### 1.3. Working Memory

Auditory and visual memory skills are enhanced in musicians and linked with early, frequent, and formal musical training [4,22,71,72,73,74,75,76,77,78,79,80,81,82,83,84,85,86]. In rare cases, no difference is documented [55] between musicians and non-musicians. A meta-analysis reported a medium effect on short-term working memory with musicians being better. The advantage was large with tonal stimuli, moderate with verbal stimuli, and small or null with visuospatial stimuli [82]. This points to an auditory-specific working memory advantage rather than a more general one. Working memory improves due to auditory processing being enhanced through music education; hearing improves cognition.

### 1.4. Frequency Discrimination

During speech processing, the pitch has hyper-linguistic characteristics that provide information on emotion and intent [87] as well as linguistic characteristics. Musicians outperform non-musicians [13,22,65,69,84,88,89,90,91,92,93,94]. This advantage was hypothesized to be a contributing factor in better speech-in-noise perception found in musicians [22]. Classical musicians were reported to have superior frequency discrimination abilities when compared to those with contemporary music (e.g., jazz, modern) background [95]. To our knowledge, there is no study researching possible differences across different musical styles that include Byzantine music.

### 1.5. Different Music Styles and Instruments

The musicians’ groups selected for the present study differ in styles and music training. Byzantine music (BM), or Byzantine chant (BC), is the traditional ecclesiastical music of the Orthodox church. It is vocal music sung by one or more chanters [96] always having a monophonic character based on eight modes (“echos”) [97]. The chanters are usually male and there is no musical instrument involved apart from the human voice [98,99]. This is in contrast with Western classical music that is polyphonic, frequently including male and female voices in the presence of instruments. Percussionists are vastly trained in rhythmic skills and timing physical flexibility and in this research are experienced in both tuned and untuned percussions.

The ordinary tuning system for Western music is the 12 equal temperament tuning system which subdivides the octave interval into 12 tones (semitones) [99]. By contrast, the BC tuning system divides the octave into 72 equal subdivisions or “moria”, according to the Patriarchal Music Committee (PMC) [100]. In comparison to Western music, where the octave is based on 12 equal units (semitones), BM has each semitone corresponding to 6 moria [96]. The elementary tone (a minor second) consists of 100 logarithmically equal micro-intervals called cents; thus, the octave consists of (12 semitones × 100 cents) 1200 cents [101]. PMC’s musician experts indicate [100] that the less audible music interval is considered to be 1 m or 16.7 c, a critically smaller interval relevant to classical music. Likewise, Sunberg [102] argues that an interval of 20 cents (1.2 moria) is hardly heard by a listener. In BM, each micro-interval differs from its neighbors by at least 2 moria [99] and the frequency steps made in Byzantine music, compared to Western music, may vary from even 1 Hz in the bass voice range.

In the literature, superior auditory processing abilities are documented for musicians on speech in noise recognition, temporal resolution, frequency discrimination, and working memory. This auditory advantage in musicians is possibly better explained as a result of neuroplasticity through music training rather than better genes as shown by previous research [103]. Could it be that this neuroplasticity is influenced by different instruments and/or musical styles? This study aims (i) to evaluate possible differences across different instrument musicians and/or different musical styles. Could different musical instruments or styles of training lead to enhanced auditory processing in specific elements that might not be the same across different style of musicians? If this is proven to be the case, it would provide more insights toward more individualized rehabilitating approaches for individuals with deficits in auditory processing. A secondary aim is to to verify that musicians have better auditory processing skills compared to non-musicians, specifically when examined with the auditory processing diagnostic test battery used in the Greek population.

## 2. Material and Methods

### 2.1. Participants

In the present study, 36 Greek professional musicians participated, divided into three groups according to specialization: 12 in byzantine music (four females), 12 in Western classical music (seven females), and 12 in percussion (four females). Musicians were performing music at a professional level with at least 10 years of musical experience (*M* = 27.58, *SD* = 10.83). Classical musicians specialized in different kinds of instruments (3 guitarists, 2 pianists, 1 clarinetist, 2 flutists, 2 violinists, 1 classical singer, and 1 trumpetist) apart from percussion. Percussionists, on the other hand, specialized both in tuned and untuned percussion. The control group comprised 12 non-musicians (10 females). The non-musician group did not get any formal music education apart from music lessons in primary and secondary mainstream education. The three experimental groups and one control group did not differ in average age (Table 1). All participants were selected by word of mouth, via Facebook posts and did not have a diagnosed neurological, language, or attention disorder as per their report. They all signed informed consent before testing. All procedures were approved by the Ethics and Bioethics Committee of the Aristotle University of Thessaloniki (6613/14 June 2022). There were no exclusion criteria as the study had to be concluded in a limited time as part of a master’s degree and the aim was to document auditory processing in as many musicians and controls as possible.

### 2.2. Procedure

Auditory Processing Tests were administered in a randomized order and included the Speech in Babble test (SinB) [104,105], Gaps-In-Noise (GIN) [50,106], Digit Span [107], Word Recognition Rhythm Component test (WRRC) [58,59], and Frequency Discrimination Limen test (DFL). These tests were administered to all musicians and non-musicians, to assess speech perception, temporal resolution, short-term and working memory, and speech comprehension with rhythm effect. We also conducted a Frequency Discrimination Limen test (DFL) which was created based on other Frequency Discrimination Limen and Just Noticeable Difference tests [91,108,109,110]. All tests were administered to all participants in a sound-treated room via headphones (TDH-50P) at 60 dB HL through a CD player and a GSI 61 audiometer.

Pure-tone audiometry using the same audiometer and headphones was implemented for all participants evaluating frequencies 250 Hz, 500 Hz, 1 KHz, 2 KHz, 4 KHz, and 8 kHz for each ear by an ENT consultant in a sound-treated booth following otoscopy. In case any other audiological evaluation was deemed necessary it was also implemented. Forty-five of them had normal hearing as defined by a measured threshold being better equal to or better than 20 dB HL for each of the tested frequencies in each ear. Three had elevated hearing thresholds as a result of sensorineural high-frequency hearing loss (Table 2). For the auditory processing evaluation, we ensured that all participants had fully understood the given instructions, besides having successfully completed the practice items of each test, before initiating the standard test procedure. All participants were tested in a soundproof booth. Auditory Processing Tests were presented in each ear at 60 dB HL [111]. All auditory processing tests are presented at a suprathreshold level, i.e., the average everyday intensity level of human communication during running speech. For the three individuals with sensorineural high-frequency hearing loss, the average threshold was abnormal only for one of the ears, the right one, having a 1.7 dB HL deviation from the upper normal limit. This being the case, the intensity at which the auditory processing tests were administered was not altered.

#### 2.2.1. Speech-in-Babble

The Speech-in-Babble (SinB) test [104,112] is administered monaurally. It includes two different lists of 50 phonetically balanced bisyllabic Greek words presented in background multi-talker babble recorded at the university cafeteria during rush hour. Each word is preceded by a carrier phrase (“pite tin lexi,” i.e., “say the word”) and participants are instructed to repeat the word heard after each trial. Five signal-to-noise ratios (SNR) [+7, +5, +3, +1, and −1] are used and each SNR is applied to ten words in each list. SNRs at which 50% of the items are correctly identified are calculated using the Spearman–Karber formula [112]. Poorer performance is reflected in higher scores (measured in dB SNR).

For each participant, two scores were calculated for each ear based on one administration of the SinB. Word-based scores SinB_RE_words and SinB_LE_words (for right and left ears respectively) and syllable-based scores SinB_RE and SinB_LE (for right and left ears respectively). For word-based scoring, correctly identified bisyllabic words are provided by the number of items in the Spearman–Karber formula. If the participant repeats only one of the two syllables presented, that word is scored as incorrect. For syllable-based scoring, the number of items in the Spearman–Karber formula is based on the number of correctly identified individual syllables. Therefore, if one syllable of the bisyllabic word is correctly recognized, the particular syllable is scored as correct and the non-recognized syllable as incorrect.

#### 2.2.2. Gaps-in-Noise

The Gaps-in-Noise (GIN) test [50,106] is administered monaurally with a different list of approximately 30 trials for each ear. A practice session of 10 trials is given before the main test. Each trial consists of 6 s of white noise with a 5 s inter-trial interval. Each broadband noise segment contains 0 to 3 gaps (silent intervals), the location of which varies. The duration of the gaps is: 2, 3, 5, 6, 8, 10, 12, 15, and 20 msec and each gap is presented six times during the test. Participants are told to indicate when they detect a gap by knocking their hands on the table. The gap detection threshold is calculated per ear as the shortest gap duration detected on at least four out of six gaps with consistent results for larger gaps. False positives are noted and subtracted from the correct responses as follows: total score = (total number of correct responses- false positives)/the number of trials in the list.

#### 2.2.3. Digit Span

Digit Span is a working memory test that involves the evaluation of both short-term memory and working memory binaurally. It consists of two different subtests with an increasing number of digits in a set of two per the same number of digits. In the first one participants have to repeat digits heard following each trial, the start off is with two digits. On the second one participants have to repeat digits heard in a backward manner (e.g., “2-9-4-6” is heard and the correct answer is “6-4-9-2”). Testing is ceased when a participant gives an incorrect answer on two trials of the same length or when all trials are exhausted. The test’s result is measured by adding the number of items correctly identified for each subtest per subtest and in total.

#### 2.2.4. Word Recognition–Rhythm Component (WRRC)

Word Recognition–Rhythm Component test [58,59] evaluates the speech in noise perception rhythm benefit. It uses three different lists of words in noise with a preceding 1 KHz beats of four sequences in each word. There are three conditions: Rhythm, Unsynchronized, and Non-Rhythm (RH, UnSc, and NR respectively). Rhythm (RH) condition: The sequence used in the RH condition is isochronous and synchronized with the following word. Unsynchronized (UnSc) condition: The sequence used in the UnSc condition is isochronous and the word is not synchronized to it. Non-rhythm (NR) condition: The sequence used in the NR condition was not rhythmic (i.e., non-isochronous). To avoid learning effects that might potentially result in some kind of rhythm perception, several sequences were used in a cyclic order, i.e., first A, then B, then C, etc. The test’s result is measured by adding the number of items correctly identified per each condition (for syllables, 16 maximum; for words, 32 maximum).

#### 2.2.5. Frequency Discrimination Limen

Frequency discrimination limen is assessed for three different frequency regions (500, 1000, and 2000 Hz). The frequency step varies from 2 Hz to 50 Hz with changes occurring every second between a standard (S) pure tone and a roving (R) pure tone (i.e., S-R-S…, etc.). Roving pure tones are randomized (Table 3). Stimuli are presented binaurally. Participants respond by knocking their hands on the table. The minimum frequency difference in Hz participants perceive is the reported threshold.

### 2.3. Statistical Analysis

All statistical analysis was conducted with SPSS Statistics 27.0, manufactured by IBM in New York. Results were evaluated for normal distribution by calculating the z values [113,114]. Tests executed were *t*-test, Mann–Whitney, Kruskal Wallis, ANOVA, and post-hoc analysis with Bonferroni correction depending on normal or non-normal distribution of variables. The significance level was set at 5% (i.e., *p* < 0.05).

## 3. Results

### 3.1. Word Recognition Rhythm Component

A one-way between-subjects ANOVA was executed to compare the effect of music experience, or its absence, on WRRC_RH2 for musicians of classical music, byzantine music, percussionists, and non-musicians. This variable was normally distributed.

**Figure 1:**
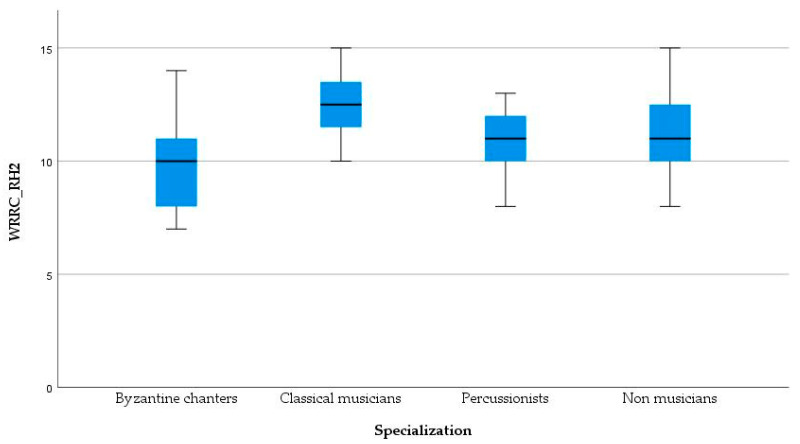
Boxplot of WRRC_RH2 by Specialization [*F*(3, 44) = 4.180, *p* = 0.007]. The median score of second syllable recognition in rhythmic conditions (RH2 scores) shows that classical musicians are better than byzantine chanters in using the rhythm effect to better perceive words in noise (specifically the second syllable of a word). WRRC_RH2 = recognized 2nd syllables in rhythm condition in WRRC test.

A significant difference in WRRC_RH2 recognition for the four groups [*F*(3, 44) = 4.180, *p* = 0.011] was found. Post hoc comparisons using the Bonferroni correction indicated that the mean score of classical musicians (*M* = 12.42, *SD* = 1.56) was significantly better than that of byzantine musicians (*M* = 9.83, *SD* = 2.20), *p* = 0.007 (Table 4, Figure 1).

The analysis did not reveal any statistical differences between four groups of musicians for the WRRC_RH1 [*H(3)* = 5.66, *p* = 0.129], WRRC_UnSc1 [*F*(3, 44) = 1.92, *p* = 0.14], and WRRC_UnSc2 [*F*(3, 44) = 2.449, *p* = 0.076] scores.

For the Speech in Babble test (SinB_RE and SinB_LE, for right and left ear respectively), an ANOVA test was conducted, to compare the three groups of musicians and non-musicians. Results did not reveal a statistically significant difference among the four groups for SinB_RE [*F*(3, 44) = 0.672, *p* = 0.574], nor for SinB_LE [*F*(3, 44) = 0.599, *p* = 0.619] (Table 5).

### 3.2. Gaps in Noise

For the Gaps in Noise test (GIN_RE and GIN_LE, for right and left ear respectively), an ANOVA test was conducted, to compare the four groups. Results did not reveal a statistically significant difference for GIN_RE [*F*(3, 44) = 0.516, *p* = 0.673], nor for GIN_LE [*F*(3, 44) = 0.248, *p* = 0.863] (Table 6).

### 3.3. Digit Span

For the Digit Span Forward (DigitF) test or the Digit Span Backwards (DigitB) test there was no statistically significant difference between musical specialization groups [*F*(3,44) = 0.709, *p* = 0.552] and [*H*(3) = 5.49, *p* = 0.13], respectively.

### 3.4. Frequency Discrimination Limen

**Figure 2:**
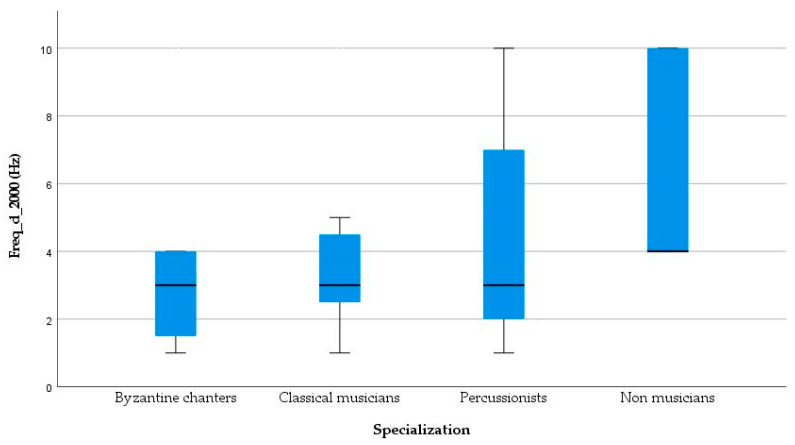
Boxplot of DFL_2000. The black lines indicate the median scores (in Hz) of Frequency Discrimination Limen at 2000 Hz for the musicians and non-musicians groups.

An ANOVA test was conducted for Frequency Discrimination Limen at 500 Hz (Freq_d_500) and did not reveal a statistically significant difference between groups (*p* = 0.678). As the criteria for normal distribution were not satisfied, the Kruskal Wallis test was administered for Frequency Discrimination Limen at 1000 Hz (Freq_d_1000) and 2000 Hz (Freq_d_2000). A statistically significant difference was not revealed for Freq_d_1000 [*H*(3) = 3.74, *p* = 0.29] between four groups of participants. Comparisons of frequency discrimination across groups found that the three groups of musicians had lower thresholds (better discrimination) at a statistically significant level [*H*(3) = 11.28, *p* = 0.010] for the 2000 Hz frequency region compared to non-musicians, as shown in Figure 2. Byzantine musicians achieved a better (lower) threshold (*M* = 3.17, *SD* = 2.44, *p* = 0.002), following classical musicians (*M* = 3.67, *SD* = 2.38, *p* = 0.017) and then, percussionists had better threshold (*M* = 4.42, *SD* = 3.45, *p* = 0.015) than the non-musicians (*M* = 6.50, *SD* = 3.09).

### 3.5. Word Recognition Rhythm Component for Musicians and Non-Musicians

A Mann-Whitney U test indicated that WRRC_RH1 scores were significantly greater for 36 musicians (*Mdn* = 27.15) than for 12 non-musicians (*Mdn* = 16.54), *U* = 120.5, *p* = 0.019 (Table 4).

Another two-sample t-test was performed to compare musicians (*M* = 13.56, *SD* = 1.297) to non-musicians (*M* = 12.58, *SD* = 1.379). Musicians showed significantly better word recognition [*t*(46) = 2.214, *p* = 0.032, *r* = 0.34), 95% CI (0.09, 1.86)], for the WRRC_UnSc1.

### 3.6. Digit Span for Musicians and Non-Musicians

A statistically significant difference was not revealed for the Digit Span Forward (DigitF) test between musicians (*M* = 10.44, *SD* = 2.04) and non-musicians (*M* = 10.00, *SD* = 2.132); *t*(46) = 0.64, *p* = 0.522).

**Figure 3:**
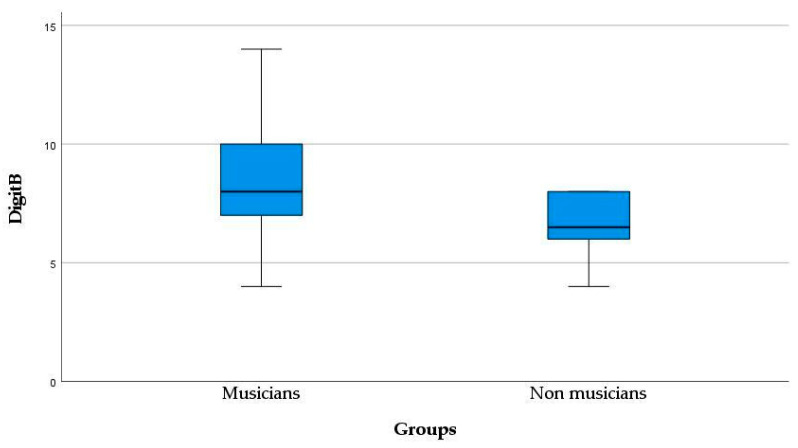
Boxplot of DigitB for musicians and non-musicians. The black lines indicate the median scores on Digit Span Backwards, according to musical engagement (*U* = 123.0, *p* = 0.025).

A Mann-Whitney U test was conducted to determine whether there is a difference in Digit Span Backwards (DigitB) scores between musicians (*Mdn* = 8.00, *SD* = 2.28) and non-musicians (*Mdn* = 6.50, *SD* = 2.02). The results indicate a significant difference between groups (*U* = 123.0, *p* = 0.025), with a working memory advantage for musicians. Overall, we conclude that there is a difference in working memory (DigitB) score between musicians and non-musicians, as shown in Figure 3.

### 3.7. Frequency Discrimination Limen for Musicians and Non-Musicians

A Mann-Whitney test for the musicians and non-musicians groups indicated significantly better frequency discrimination (*U* = 82.50, *p* = 0.001) for musicians in general (*M* = 3.75, *SD* = 2.77) compared to non-musicians (*M* = 6.50, *SD* = 3.09).

## 4. Discussion

This study’s primary aim was to assess auditory processing among musicians of three different styles, Byzantine music, Western classical music (melodic instruments), and percussion (tuned and untuned). Any documented differences might provide insight into individualized rehabilitation through specific styles of musical education and training. A secondary aim was to compare musicians with non-musicians in the Greek population and verify the musicians’ advantage in auditory skills supported in previous research [22,52,65,115,116].

The present study shows a Western classical musicians’ advantage in speech in noise recognition of the second syllable with a good use of the rhythm effect, compared to Byzantine musicians. This result is novel as there are no other studies researching auditory processing in Byzantine chanters. It provides information on different levels of improvements regarding auditory processing as a result of music education depending on the style of music. The clinical implications of this result are more in favor of a specific tailored auditory training as a rehabilitation tool, as opposed to a one, fits all approach that is the usual case with software available auditory training.

Musicians’ better performance in various auditory processing components was verified. They were better in word-in- noise recognition in all three conditions (rhythmic, unsynchronized, and non-rhythmic) in the WRRC test with the Western Classical musicians being the best compared to the other two groups in the non-rhythmic component. The results of the present study show that the enhanced performance of speech in noise perception in the WRRC test is most probably not due to a rhythm advantage but due to the musicians’ ability to not be easily distracted by other asynchronous stimuli.

Byzantine chanters were found to be better at the Frequency Discrimination Threshold for 2000 Hz compared to the other two groups of musicians. This result is novel as it concerns Byzantine music, and it may be due to the different tuning system compared to Western music with each semitone corresponding to 6 moria subdivides. Of interest, previous research revealed that vocalists have better frequency discrimination than non-musicians [117]. Byzantine chanters also had a better working short-term memory for the backward subtest compared to the other musicians.

However, for speech recognition in the SinB test, as well as, for temporal resolution in the GIN test, there was no significant difference among musicians from the three different styles and non-musicians. The non-musicians’ control group displayed exceptionally good results comparable to other studies on musicians’ outcomes [60].

Results on the WRRC test state that musicians can perceive speech in noise in any condition thus not supporting a specific rhythm effect. They appear to be better at an untrained auditory processing component regardless of the presence of rhythm. However, the advantage of classical musicians compared to the other group of musicians in this study for the second syllable discrimination in noise indicates that there is a rhythm effect benefit for this group of musicians. Enhanced frequency discrimination in musicians revealed in the present study is in accordance with recent research highlighting the presence of a correlation between frequency precision and Goldsmiths Musical Sophistication Index that is specific to the auditory domain and is unrelated to vision or amplitude modulation [8]. The 2000 Hz specific frequency discrimination advantage shown by Byzantine chanters in our study should be further investigated in a larger sample. The working memory advantage documented in the more difficult part of the digit span test (backward subtest) is expected when comparing musicians versus non-musicians.

The fact that musical practice takes many forms, and it is yet unknown which specific elements of musical experience or expertise may direct speech perception advantages, may be a potential contributing component in the mixed experimental outcomes with musician versus non-musician comparisons. Even within categories such as classical or jazz performance, there is great diversity in instruction methods (e.g., learning to play from a score vs. learning to play by ear) that may influence the development of specific aspects of musical competence, such as rhythm perception and auditory memory [52].

Moreover, there is a difference observed in beat alignment and the time spent on musical practice [118]. Studies in musicians have shown that the more years a person is intensively engaged in music, the more areas of the brain are involved in perceiving, analyzing, and recording it, and the more neural networks develop to convey the language of music to wider areas of the brain and make it “musically driven”. Music contributes to the development of many skills and to the activation of many brain centers, which are associated with cognitive functions [98].

Slater et al. [52,119] attempted to estimate which specific rhythmic skills are associated with speech perception in noise, and whether these relationships extend to measures of rhythm production as well as perception. In that research, percussionists outperformed non-musicians, apart from speech-in-noise perception, on sequence- and beat-based drumming tasks. The speech-in-noise perception was correlated with the two sequence-based tasks (drumming to metrical and jittered sequences) [119]. Percussionists and singers did not differ in their performance on the musical competence test (rhythm or melody subtests), speech-in-noise perception (words or sentences), or auditory working memory [52]. Cognitive factors such as memory intervene unquestionably in the relationships between musical skill and hearing speech in noise [15]. The ability to perceive words in noise (WIN) did not relate to either rhythmic or melodic competence, nor to working memory competence [52]. Interestingly, recent studies substantiated enhanced pitch perception in musicians and melodic discrimination yet did not detect any advantage for speech-in-noise comprehension [52,57].

Although the present study cannot speak to the precise effects of music training in distinct musical styles, our cross-sectional findings provide a basis for further investigation into the potential for music training to reinforce auditory processing skills and building blocks of communication. In parallel, music therapy is the clinical application of music to treat disease in individuals who can benefit from music and thereby improve their quality of life. It is known since the Byzantine era when many of the hospitals of Constantinople applied music therapy to neurological patients [120]. Music therapy is a really pleasant and painless therapeutic method, usually practiced by specialized therapists, who have the appropriate knowledge and experience [121]. Therefore, potentially, we could suggest that for (C)APD cases music training, or music therapy, apart from auditory training, could be a pleasant and effective way to sharpen auditory skills.

Further research ideas have to do with seeking differences in Frequency Discrimination Limen among Byzantine chanters and other musical genres. Moreover, a fertile area of research could involve Byzantine chanters, other vocalists, or musicians specialized in other musical types of instruments (e.g., woodwinds, brass, strings), as well as their relation to speech in noise comprehension. Likewise, studies applying music training for auditory processing, setting age limits, and hearing sensitivity directions, could supplement recommendations, as a way to detect the best approach to train auditory processing disorders. Moreover, examinations with non-psychometric tests, like fMRI, would be an interesting extension of the research and its results.

Among the limitations of the present study is the absence of data on the educational profiles of participants. Secondly, although there was no statistical evidence for participants’ mean age, a slight difference in age among the four groups, could be the reason for the absence of differentiation among the three musical groups, or from musicians to non-musicians, in speech in noise comprehension, according to previous research [122,123]. Our subjects were also not required to have pure-tone thresholds better than 20 dB at the audiometric test or fill a questionnaire to assess the impact of their hearing impairment [124], whereas normal hearing was necessary for Varnet et al. [125] as musicians are more likely to experience hearing problems [124,125,126]. Interestingly, out of the three musicians with abnormal pure tone thresholds in one or two high frequencies, only one has an average pure tone threshold of more than 20 dB, which is considered normal. It should be noted that the musician with an average hearing threshold of 20 dB, exceeds it by less than 2 dB HL. As the average pure tone threshold of audiometric tested frequencies is used for auditory processing evaluation determination of needed adjustments, there was no need for any adjustment of intensity when administering the auditory processing test battery for any of the participants in this study. Finally, participants were not matched by gender and musical instruments, for musicians. A possible female auditory processing advantage [127] could not explain the reported better performance in musicians in the present study as our musicians’ groups include more men than women compared to the control group.

## 5. Conclusions

The study reveals different auditory processing elements enhancement as a result of different training in different musical styles. Neuroplasticity seems to be specific while extrapolating to non-trained elements of auditory processing such as speech in noise perception. This sets the basis for individualized auditory training in individuals with APD.

## Figures and Tables

**Table 1:** Mean and SD for participants’ age. ANOVA did not reveal a statistically significant difference in the average age of the four categories of participants [*F*(3, 44) = 0.926, *p* = 0.436].

	Age
Byzantine chanters	39.17 (13.361)
Classical musicians	37.92 (11.866)
Percussionists	32.75 (10.635)
Non-musicians	33.25 (10.678)
Total	35.77 (11.661)

**Table 2:** Pure tone thresholds for 3 participants with mild high-frequency sensorineural hearing loss. Hearing Threshold (H. Thr.) for Left Ear (LE) and Right Ear (RE). The other frequency thresholds tested were normal, thus they are not presented in Table 2.

Frequency/Ear	4 kHz LE	8 kHz LE	Average H. Thr. LE	4 kHz RE	8 kHz RE	Average H. Thr. RE
Participant 1	40 dB	40 dB	16.7 dB	45 dB	40 dB	21.7 dB
Participant 2	30 dB	20 dB	12.5 dB	30 dB	20 dB	9.2 dB
Participant 3	45 dB	0 dB	7.5 dB	45 dB	20 dB	13 dB

**Table 3:** Frequency Discrimination Limen procedure example, for three parts. “S” is for standard pure tone and “R” for roving pure tone.

	S	R	S	R	S	R	S	R	S	R
500 Hz (part1)	500	530	500	510	500	504	500	520	500	501
1000 Hz (part2)	1000	1002	1000	1050	1000	1020	1000	1004	1000	1003
2000 Hz (part3)	2000	2005	2000	2020	2000	2003	2000	2010	2000	2001

**Table 4:** Mean scores (SDs in parenthesis) for each group on 1st (RH1) and 2nd (RH2) syllable of WRRC on rhythmic condition.

	RH1	RH2
Classical musicians	13.33 (1.30)	12.42 (1.56)
Byzantine musicians	13.17 (1.11)	9.83 (2.20)
Percussionists	13.17 (1.33)	10.92 (1.37)
Non-musicians	12.25 (.96)	11.33 (1.96)

**Table 5:** Mean (SD) scores for Speech in Babble for the Right and Left ear, respectively.

	SinB_RE	SinB_LE
Classical musicians	−0.18 (0.36)	−1.23 (0.23)
Byzantine chanters	−0.08 (0.53)	−1.20 (0.19)
Percussionists	−0.30 (0.37)	−1.14 (0.28)
Non-musicians	−0.28 (0.39)	−1.11 (0.28)

**Table 6:** Mean (SD) scores in msec for the GIN test.

	GIN_RE	GIN_LE
Classical musicians	5.33 (1.43)	5.67 (1.23)
Byzantine chanters	5.50 (1.31)	5.83 (1.46)
Percussionists	5.50 (1.67)	5.58 (1.31)
Non-musicians	4.92 (0.66)	5.42 (0.66)

## Data Availability

The data presented in this study are available on request from the first author.

## Abbreviations

APD: auditory processing disorder, SNR: signal-to-noise ratio, dB: decibel, RE: right ear, LE: left ear, BM: Byzantine music, BC: Byzantine chant.
